# Short-Term Memory Effects on Crossing the Boundary: Discrimination between Large and Small Quantities in Angelfish (*Pterophyllum scalare*)

**DOI:** 10.1371/journal.pone.0162923

**Published:** 2016-09-28

**Authors:** Luis M. Gómez-Laplaza, Robert Gerlai

**Affiliations:** 1 Department of Psychology, University of Oviedo, Oviedo, Spain; 2 Department of Psychology, University of Toronto Mississauga, Mississauga, Canada; Universidad de Chile, CHILE

## Abstract

Rudimentary quantification abilities are found in numerous animal species and in human infants all demonstrating the ability to discriminate between quantities differing in numerical size. An open question is whether individuals rely on different underlying systems to discriminate between large (analogue magnitude system (AMS) for number of items exceeding 3) and small quantities (object-file system (OFS) for number of items below 4), or they use only one system (AMS) for the entire number range. The two-system hypothesis has been supported by finding reduced ability to discriminate between quantities that cross the large-small boundary in several species. Recently, the role of cognitive representation, i.e., memory, in quantity discrimination has also been recognized. Here, we investigated whether angelfish can discriminate quantities across the boundary under two memory conditions. In a binary choice test, single angelfish were allowed to see groups (shoals) of conspecifics of different numerical size on the two sides of their test tank. In Experiment 1, their choice was recorded after a 2-sec retention interval during which shoal size information was unavailable. Angelfish were able to discriminate the larger shoal across the boundary when the shoals differed by a 2:1 or higher ratio, but not when the ratio was lower. In Experiment 2, however, with a 15-sec retention interval, angelfish could only detect a four-fold difference in ratio but failed to detect a three- or a two-fold difference across the boundary. These results suggest that angelfish can remember smaller differences for a short (2 sec) but not for a longer (15 sec) period. Together with previous findings, the current results support the idea that angelfish use two distinct systems for representing quantity, but they may recruit the AMS even for the small number range under some circumstances, e.g., when higher memory demand is imposed by a greater retention interval.

## Introduction

For the last decade, an intense debate has been taking place over the mechanisms of the cognitive representation underlying discrimination between quantities. Two main non-verbal representational systems have been proposed, initially to account for findings in human research [1,2]. According to some investigators, small sets of items (< 4) may be represented by a precise mechanism usually named the object-file system (OFS). Whereas an approximate mechanism, named the analog magnitude system (AMS), may be used to represent large sets of items (≥ 4). The OFS is viewed as a mechanism of visual attention, rather than a proper number system [3], with a limited capacity because of visual attention restricts the number of object files available. Using this system, discrimination is performed via one-to-one correspondence between the components of the contrasted sets up to the capacity limit of about 3 or 4 items [4–6]. In contrast, under the AMS the discrimination of two large quantities is determined by their ratio, rather than their absolute numerical difference. A characteristic of the AMS is that representations of the magnitudes are only approximate and accuracy decreases as the ratio between quantities approaches 1:1, i.e., the change in accuracy follows Weber’s law [7,8].

The idea of the two systems has been proposed because human infants’ number discrimination appears to follow Weber’s law for the discrimination between large numbers, but limited by absolute set size when small number of items is being discriminated [5,9,10]. However, other findings suggest that the AMS may also account for the successful discrimination of small sets, i.e., for processing the entire number range, a notion that questions the existence of OFM [7,11,12]. Thus, the question of whether one system or two systems are required for quantity discrimination is currently disputed, and empirical support for both views has been found in human infants (see [13,14]). Another, emerging, view is that perhaps small quantities may be represented by both the OFS and the AMS. Proponents of this view suggest that the experimental circumstances, including stimulus features, task demands, heterogeneity of the stimuli, or certain non-numerical properties of the stimuli, may determine which system is engaged in the discrimination (e.g., [14,15]).

One of the main findings favoring the existence of the two systems is the so-called ‘boundary effect’. This effect has elicited some attention in research because of the infants’ repeated failure to discriminate sets across the large-small boundary. That is, infants were able to discriminate between small sets: 2 vs. 1, 3 vs. 1, and 3 vs. 2 items, but they surprisingly failed to discriminate between contrasts in which one set belonged to the large and the other to the small number range, i.e., when the contrasted sets crossed the boundary (e.g., contrasts 4 vs. 1, or 4 vs. 2) [2,4,5]. This failure has generally been interpreted as indicating that infants are not representing large and small numbers within a single system, and that the impairment in the discrimination may reflect that items are represented using distinct systems. Thus, a conflict between representations impeded comparison [6,11].

A similar controversy about the representational systems engaged in the discrimination of large and small sets surrounds studies on quantity discrimination in nonhuman animals. However, substantial evidence favours representation of quantities based on AMS, and results in a diversity of studies with nonhuman animal species indicate that the discrimination of both large and small sets involves such a system (reviewed in [16,17]). In contrast, only limited amount of evidence exists suggesting the involvement of the OFS in the discrimination of small sets, and thus the operation of the OFS to represent small quantities has been questioned [18,19]. Nevertheless, similarly to human infants, in nonhuman animals failure to discriminate contrasted item sets in cross-boundary comparisons has been suggested as evidence of the existence of the two separate systems [20–23].

Specifically in fish, the issue regarding the existence of one or two distinct quantity mechanisms has not been resolved and is rather equivocal. Some findings appear to support the existence of two systems, and suggest the engagement of the OFS with an upper limit in quantity discrimination of about 3–4 elements, e.g., in goldbelly topminnows and mosquitofish [24,25]. Similarly, some studies with guppies favor the two-system hypothesis and the deployment of the OFS for discriminating small quantities [26–28]. However, other findings in diverse fish species support the idea of the use of a single AMS to represent both large and small elements. For example, Mehlis et al. [29] studying three-spined sticklebacks found that numerically different shoals were discriminated in a manner that followed Weber’s law even within the small number range. Challenges to the concept of two distinct mechanisms also come from results with other species including the blind cavefish [30], the redtail splitfin fish [31] and the zebrafish [32]. These studies all suggest discrimination abilities to follow Weber’s law, and contradict the involvement of a separate OFS for discrimination of small quantities. Even in a study with guppies that examined individual differences in quantification abilities, performance was better explained by an AMS operating over the entire numerical range [33]. Nevertheless, as in human infants, it has been suggested that in fish too both discrimination systems may exist, and contextual variables (e.g., type of stimuli, or other idiosyncratic task features and methodology employed) may favor the involvement of one versus the other system [34].

In previous studies, we have demonstrated that angelfish (*Pterophyllum scalare*), apparently seeking protection from potential threats in a novel environment, exhibited a preference for the larger of two shoals when the contrasting shoals were fully visible and composed of a small number of members, i.e., less than 4 [35]. As subjects were able to discriminate 3 versus 2, 3 versus 1, and 2 versus 1 with similar accuracy, but failed to discriminate 4 versus 3 individuals, the results suggested that angelfish accomplished the task employing an OFS with an upper limit of three individuals. In another study with shoals now consisting of a relatively large number of members [36], we found that angelfish followed a ratio-dependent performance, a pattern consistent with the existence of an AMS. Likewise, we have repeatedly shown successful quantity discrimination between small shoals (3 vs. 2 conspecifics), as well as between large shoals when the number of members in the shoals changed two-fold (10 vs. 5 conspecifics) [37–39]. In the latter studies, we also found that the role played by certain continuous variables characterizing shoal size was dependent upon whether numerically large or small shoals were involved in the discrimination. These findings suggested the potential engagement of different processing systems for the discrimination of large versus small quantities in angelfish. Notably, in some of the above studies, angelfish were able to discriminate between shoals of numerical size that crossed the large-small boundary, leaving open the question of the existence of one or two discrimination mechanisms. Another interesting question that arose in the studies investigating numerical discrimination abilities in human and non-human animals has been whether the sets of items the subject discriminates are represented in memory or whether they need to be observable at the time of making a choice [40,41].

Recently, we have adopted a new procedure, modified from that developed by Stancher et al. [31]. By this procedure the conspecific stimulus shoals were not fully visible during tests but only one angelfish of each stimulus shoal was visible to the subjects. Thus, although during a pretest period the stimulus shoals were fully visible at the opposite ends of the test aquarium, during the actual choice test the experimental subject had to rely on its memory of the location of the previously seen shoals. Similar, memory-based, procedures have been employed with other species, including human infants [4,5,20,41–43]. We have found angelfish to be able to rely on their visual short-term memory when discriminating shoals both within the small [44] and the large number range [45]. Furthermore, the performance of angelfish under this memory demand was found similar to that when the stimulus shoals were fully visible during tests [44,45]. These studies, however, did not specifically examine whether different mechanisms were involved in the discrimination of large versus small shoals and whether different memory demands (length of memory) differentially affect performance in the large versus the small number range.

In the present study, in order to gain a better understanding of the hypothetical system(s) underlying quantity discrimination in angelfish, we specifically investigated the boundary effect employing the above mentioned memory-based discrimination procedure. By presenting contrasts across the large-small boundary, in this study we investigated the existence of one versus two systems. In addition, we also explore the effect of working memory on performance by systematically controlling the memory demand of the task, i.e., by varying the retention interval between pretest and test from 2 sec (Experiment 1) to 15 sec (Experiment 2).

## Materials and Methods

### Ethics statement

The experiments described here comply with the current law of the country (Spain) in which they were performed, and were approved by the Committee on the Ethics of Animal Experiments of the University of Oviedo (permit number: 13-INV-2010).

### Subjects and housing conditions

Wild type juvenile angelfish (*Pterophyllum scalare*, about 3.0–3.3 cm standard length) were obtained from local commercial supplier Pajarería Amazonas S.L. (Oviedo, Spain) that receive their fish from Avi-Piscícola del Norte S.L. (Irún, Guipúzcoa, Spain). Only juveniles of this sexually monomorphic species were studied in order to eliminate possible confounding effects arising from courtship or agonistic interactions. The fish were housed in glass holding aquaria (length × width × depth: 60 cm × 30 cm × 40 cm) in groups of 18–20, and were allowed a minimum of a 2-week acclimation period prior to testing.

Test fish and stimulus fish (which were used to elicit test fish behaviour) were randomly selected and kept separately, with no visual and olfactory communication being possible between fish in the separate aquaria. Aquaria were maintained under standardized conditions with dechlorinated tap water kept at 26 ± 1°C using thermostat-controlled heaters. Each aquarium was illuminated by a 15-W white fluorescent light tube placed above the tank and a 12:12-h light:dark cycle was maintained with lights on at 08.30 hour. External filters continuously cleaned the aquaria, which had a 2-cm deep gravel substratum. The fish were fed commercial fish food (JBL GALA, JBL GmbH & Co. KG, Neuhofen, Germany) twice daily, at 10.00 h and at 18.00 h.

### Experimental apparatus

**Fig 1:**
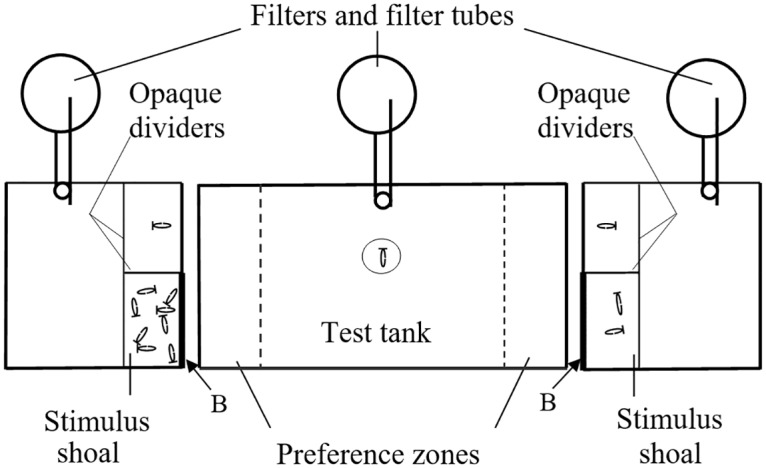
The experimental apparatus. Top view of the experimental apparatus showing the central test aquarium and the two stimulus aquaria, one on each side of the test aquarium. In the stimulus aquaria, opaque dividers were used to isolate a 10-cm compartment where the stimulus shoals were placed. An additional opaque piece divided this compartment in half: in one half, a stimulus angelfish was placed, and in the other the rest of the members of the stimulus shoals. Opaque barriers (B) were used to hide the stimulus shoals from the test fish during the pretest phase (for 2 sec in Experiment 1, and for 15 sec in Experiment 2). Then, the barriers were moved backwards so as to present a single stimulus fish on both sides while the rest of the members of the stimulus shoals was kept hidden. The time the test fish spent within the 10 cm of the stimulus (preference zones) was recorded. Modified from [45].

The experimental apparatus was similar to what we used in previous studies (e.g., [45]). It consisted of a test aquarium, identical in all respects to the holding aquaria maintained under the same conditions, with one stimulus aquarium positioned at each end of the test aquarium (see Fig 1). The stimulus aquaria (length × width × depth: 30 cm x 30 c m x 40 cm) had a matching sized side (30 cm x 40 cm) with the short lateral side of the test aquarium. An opaque divider isolated a 10-cm compartment in the stimulus aquaria where the stimulus shoals were placed. An additional opaque divider separated the stimulus compartment in two equal independent parts facing the test aquarium. To prevent the fish from being disturbed by external visual stimuli, all exterior walls of the aquaria that were not adjacent to other aquaria were lined with white cardboard, except for the front.

Six equal zones were marked in the test aquarium by five vertical lines drawn on the front and back walls. It allowed measurements of the test fish’s movements and position. The two-10 cm zones closest to the stimulus aquaria were considered to be the preference zones. Swimming activity of test fish was measured as the frequency (number of times) the fish crossed the lines drawn on the walls of the aquarium. At least three-quarters of the body length of the fish had to be within the boundary for the fish to be considered being inside a particular zone.

### Procedure

The procedure followed that of a recent study [45]. In each trial, a single test angelfish was given a choice between two numerically different shoals of conspecifics presented simultaneously and positioned in the stimulus aquaria on opposite sides of the test aquarium. The stimulus fish were chosen at random from one of the stimulus fish holding aquaria, and were gently transferred in small Perspex containers into the stimulus compartment. One fish of each of the stimulus shoals was individually placed into the rear part of each of the corresponding stimulus compartments, whereas the rest of the members of each of the stimulus shoals were placed into the front part of the stimulus compartments from the observer’ point of view (see Fig 1). The positioning of the larger versus smaller shoal was initially randomized for each test fish and subsequently counterbalanced across trials. Trials took place 15–30 min after feeding in the morning to control for possible confounding effects caused by circadian rhythm or different level of hunger [46].

Test fish were randomly selected from a test fish holding tank, and were introduced singly to the centre of the test aquarium via a transparent, open-ended, plastic cylinder (7 cm diameter), in which they were kept for 5 min. During this acclimation period of time, test fish could see the full stimulus shoals presented in the stimulus compartments at both sides of the experimental aquarium from an equal distance. When in the cylinder, all test fish oriented towards both shoals. At the end of this period, removable opaque white barriers (B in Fig 1) were placed outside the two end sides of the test aquarium to visually isolate test fish from all stimulus fish. After a short period of time (2 sec, Experiment 1; 15 sec, Experiment 2), the opaque barriers were moved backwards and placed in the front part of the stimulus compartment (B in Fig 1) leaving just one stimulus fish of each shoal (the one placed in the rear compartment from the observer’ point of view) visible for the test fish. The transparent cylinder was then gently removed and the test fish thus released to swim freely. Shoaling preference was recorded over a 15-min period and was defined as the time spent by the test fish in the 10-cm preference zones, that is, within 10 cm from the wall adjacent to the stimulus shoal aquaria on either side. Behavioural responses of the test fish were recorded with a video camera (Sony video Hi8, model CCD-TR750E) positioned 180 cm away in front of the tank concealed behind a blind. The recordings were later replayed for analysis. If test fish remember the location of the larger versus smaller shoals they are expected to approach the zone close to the previously visible larger shoal.

After each trial, the aquaria were cleaned before being replenished with dechlorinated tap water. Individual fish were tested only once, and none of the fish in the stimulus shoals were used as test fish and vice versa. Within each experiment, the order of testing was randomized according to different treatment conditions. Stimulus shoals were rearranged after each trial, so that each test fish was exposed to stimulus shoals with different individual members in them. All fish were returned to the supplier at the end of the study.

### Statistical analysis

In each experiment, the data were tested for normality (using the Kolmogorov-Smirnov one sample test) and equality of variance (using Levene’s test) before analysis. The time spent in the preference zones (sec) was considered as a measure of each test fish’s social preference for a particular stimulus. We calculated a preference index for each test fish, defined as the proportion of time test fish spent close to the larger stimulus shoal: the time spent in the preference zone near the larger stimulus shoal was divided by the total time spent shoaling (i.e., sum of the time spent within 10 cm from either stimulus shoals). Based on our previous studies conducted with angelfish and results obtained with a large number of other vertebrate species, we expected a unidirectional preference for the larger shoal. Thus, a one sample one-tailed t–test was employed to investigate whether the observed preference index was significantly (p > 0.05) higher than chance (50%). The Holm-Bonferroni sequential correction method was employed to minimise type I error [47]. A one-way ANOVA for independent samples was used to analyze the effect of the treatments on preference, and in case of a significant effect Tukey Honestly Significant Difference (HSD) post hoc multiple comparison test was performed to determine where significant differences lay. In Experiment 2, the criterion of variance homogeneity was not met for swimming activity (Levene test: *p* = 0.004), and data were log transformed before performing ANOVA and Tukey HSD post hoc test.

Last, a criterion of exclusion was applied as follows: during the binary choice test subjects had to enter both preference zones at least once, otherwise they were excluded from the experiments and replaced by another fish. In Experiment 1, four subjects (4%) were excluded and replaced, whereas in Experiment 2 seven subjects (6%) were excluded and replaced.

### Experiment 1: Discrimination across the large-small boundary with a retention interval of 2 sec

#### Method

The goal of this experiment was to determine whether angelfish prefer the larger of two shoals of conspecifics using the current task which had a low working memory demand (2 sec). Test fish matched for standard length (± 0.20 cm) with the stimulus fish were presented with eight different binary choices. The following contrasts were employed all of them crossing the divide between large (≥ 4) and small (< 4) numbers: 4 fish versus 3 fish, 5 fish versus 3 fish, 6 fish versus 3 fish, 4 fish versus 2 fish, 7 fish versus 3 fish, 9 fish versus 3 fish, 6 fish versus 2 fish and 8 fish versus 2 fish. These comparisons correspond to increasing ratios from 1.33:1 to 4:1 passing through 2:1 and 3:1 ratios. Successful discrimination of these contrasts would favour the involvement of the analogue magnitude system (AMS) for the entire number range. Alternatively, the object-file system representation may be involved for small sets of items with an upper limit of about 3–4 (e.g., [1]). The sample size of experimental fish was 12 for each of the eight sets of contrasts; thus, a total of 96 experimental fish were tested.

#### Results

**Fig 2:**
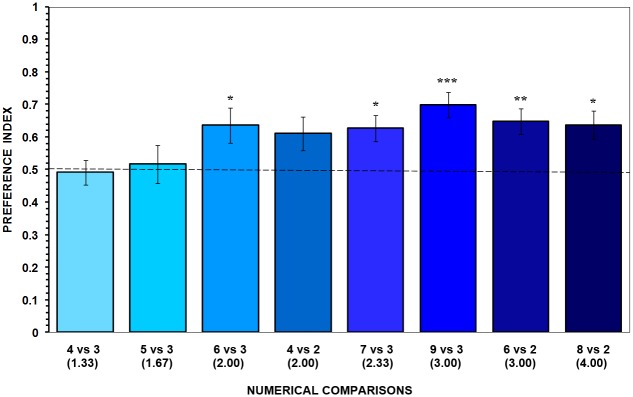
Results of Experiment 1. Mean ± SEM proportion of time test fish stayed in the 10-cm preference zone close to the larger stimulus shoal relative to the total time they stayed in both preference zones (*preference index*). Numbers in parentheses indicate the ratio of the larger to the smaller shoal. The shoal contrasts treatments are shown in increasing ratios. Values above 0.5 indicate a preference for the larger shoal. Significant departure from the null hypothesis of no preference is indicated by asterisks: *** *p* < 0.005, ** *p* < 0.01, * *p ≤* 0.05.

After breaking the visual contact with the stimulus shoals for 2 sec, angelfish generally showed preference to stay in the zone adjacent to the stimulus tank that contained the larger shoal. With the exception of the contrasts 4 vs. 3 and 5 vs. 3 where no significant preference could be detected for any of the shoals (t-test with Holm-Bonferroni correction: t_11_ = 0.215, *p* = 0.834, and t_11_ = 0.298, and, *p* = 0.385, respectively; Fig 2), in the rest of the contrasts fish showed a preference for the larger shoal. One-sample one-tailed t-test (with Holm-Bonferroni correction) revealed that experimental fish spent significantly more time on the side where the larger shoal was shown prior to the test as compared to the side where the smaller shoal was shown prior to the test (t_11_ = 2.549–5.116, *p*_*s*_ = 0.05 –< 0.004; Fig 2). Note that, in contrast with our previous results [44], no significant discrimination was detected for the contrast 4 vs. 2, as the p value only bordered significance (t_11_ = 2.134, *p* = 0.084). Nevertheless, analysis of the first choice made by the experimental fish showed that 10 out of the 12 test angelfish of this treatment chose the preference zone near the larger shoal (binomial probability test: *p* = 0.039). This finding supports the notion that fish in the 4 vs. 2 contrast did exhibit a preference for the larger shoal. Interestingly, results also showed that angelfish were able to discriminate between contrasts crossing the boundary and responded to the location of the previously seen shoals when the ratio was 2:1 or greater, whereas below that ratio they did not differentiate between locations of previously seen shoals.

A significant difference between the eight treatments in the magnitude of the preference was found by one-way ANOVA (F_7,88_ = 2.280, *p* = 0.035). This significant shoal contrast effect was due to the greater preference for the larger shoal in the contrast 9 vs. 3 relative to that in the contrasts with a ratio below 2:1 (Tukey HSD test, *p* < 0.05). These effects cannot be attributed to differential swimming activity of the fish in the different contrasts, as no significant differences were found in swimming activity across the experimental fish of the eight contrasts during testing (one-way ANOVA: F_7,88_ = 1.923, *p* = 0.075).

### Experiment 2: Discrimination across the large-small boundary with a retention interval of 15 sec

#### Method

In Experiment 1 angelfish were generally found to be able to discriminate between sets crossing the large-small number divide when the ratio was 2:1 or greater, but not below. In this latter experiment, the memory demand was low: the test fish only had to remember the previously seen shoals for 2 sec. In Experiment 2 to test the role played by visual short-term memory in the discrimination, we presented fish with a more demanding memory task by imposing a longer (15 sec long) retention interval. During this period, test fish could not see any of the stimulus shoals. Thus, fish had to be able to use mental representations (memory) of the quantities previously seen, and their locations, for a successful discrimination. The same eight contrasts crossing the large-small boundary used in Experiment 1 were employed. In addition, a contrast consisting of 12 fish versus three fish (ratio 4:1) was also included. Twelve fish were tested for each of the nine sets of choices, a total of 108 fish. The parameters recorded and the experimental procedures performed were otherwise identical between Experiment 1 and 2.

#### Results

**Fig 3:**
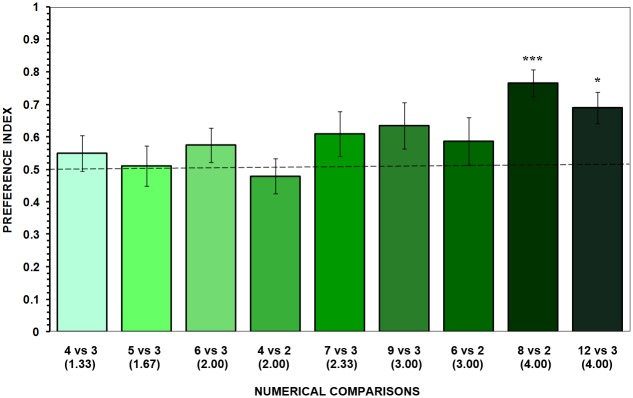
Results of Experiment 2. Mean ± SEM proportion of time test fish stayed in the 10-cm preference zone close to the larger stimulus shoal relative to the total time they stayed in both preference zones (*preference index*). Numbers in parentheses indicate the ratio of the larger to the smaller shoal, and the treatments are shown in increasing ratios. Values above 0.5 indicate a preference for the larger shoal. Significant departure from the null hypothesis of no preference is indicated by asterisks: *** *p* < 0.005, * *p* < 0.05.

When the ratio between the stimulus shoals was 3:1 or below, angelfish exhibited no significant preference for any of the two shoals in each of the comparisons (one sample one-tailed Holm-Bonferroni corrected t-tests: t_11_ = 0.162–1.870, *p*_*s*_ > 0.05; Fig 3). The failure in the discrimination of these contrasts are in disagreement with the results of Experiment 1, in which, under a less stringent memory demand, angelfish were able to distinguish contrasts down to the 2:1 ratio. Nevertheless, the results of Experiment 2 do demonstrate that under the more stringent memory demand of 15 sec retention interval angelfish are able to show preference for the larger shoal when a numerical ratio between the contrasted shoals is 4:1. That is, angelfish stayed longer in the preference zone that was adjacent to where the fish previously saw the larger shoal as compared to the opposite side where the smaller shoal used to be seen in the contrast conditions 8 vs. 2 and 12 vs. 3 (t_11_ = 6.359, *p* < 0.0045 and t_11_ = 3.877, *p* = 0.012, respectively; Fig 3).

A subsequent one-way ANOVA revealed a significant difference between the magnitude of the preferences among the nine treatment groups (F_8,99_ = 2.194, *p* = 0.034). This effect was mainly due to the greater preference for the larger shoal in the contrast 8 vs. 2 relative to that in the contrasts 4 vs. 2 (Tukey HSD test, *p* = 0.027).

Significant difference was also found between the swimming activity levels of the nine treatment groups (F_8,99_ = 2.646, *p* = 0.011). Tukey HSD test showed that the locomotor activity of experimental fish in the 8 vs. 2 contrast was significantly lower than that of fish in the contrasts 4 vs. 3 (*p* = 0.031) and 5 vs. 3 (*p* = 0.012), which moved from one side of the aquaria to the other more frequently possibly because they could not distinguish the larger from the smaller shoal.

## Discussion

A debate persists about the mechanism(s) of nonverbal representation engaged in discrimination of quantities. The controversy is mainly focused on the system underlying representation of small quantities (< 4). Some propose that an analogue magnitude system (AMS) is involved in both small and large quantities, whereas others found evidence that a separate system, the object-file system (OFS), is employed when small quantities of items need to be distinguished. In the current study, we investigated the boundary effect since failure to discriminate two quantities when one belongs to the large and the other to the small number range has been argued to be due to an incompatibility of the two representational systems, thus supporting the existence of both systems [5,11]. In our study, angelfish were not allowed to observe the whole sets (shoals of conspecifics) at the moment of choice, a procedure that is conceptually similar to what has been employed with some other species (e.g., [4,6,20,34,48,49]). This procedure forced the experimental subject to make decisions based upon mental representation, i.e., memory, of the quantities rather than upon direct visual observation of the contrasted quantities. We also investigated whether changing the length of the interval between stimulus presentation (pretest) and the start of the choice test had a differential effect on using the OFS and AMS system in angelfish.

In Experiment 1, when a retention interval of 2 sec was employed, angelfish were able to successfully discriminate across the boundary when the contrasting shoals differed by a 2:1 numerical ratio or higher. Angelfish, however, failed to discriminate between shoals that differed by a ratio below 2:1. The results showed that ratio between shoal sizes played a prominent role in the discrimination. Such ratio-dependent performance is consistent with the existence of an AMS that follows Weber’s law: discrimination is based on the numerical ratio between sets, and accuracy decreases as the ratio narrows. These findings suggest that quantity discrimination for small as well as for large number of items in angelfish is characterized by a single mechanism, the AMS. Since previously we have found a set size limit of three elements for small quantities (in agreement with an OFS), and claimed that it lent support to the existence of two underlying mechanism of discrimination in angelfish [34], the present results appear contradictory. Also, investigation of the effects of certain non-numerical, continuous, variables on quantity discrimination in angelfish revealed differential results for choice tests conducted within the small or the large number ranges ([37–39]; see also [28,50]) reinforcing the idea of two different processing systems.

Yet other studies in which angelfish could view the contrasted shoals during the choice test, found that the experimental fish could cross the large-small boundary (e.g., 4 vs. 1, 4 vs. 2) [35,36], and also when a retention interval of 2 sec was imposed [44]. In such studies, successful quantity discrimination across the boundary occurred when the number of members in the contrasted shoals differed by at least twofold. Our current results also show that a 2:1 or greater ratio between the larger and the smaller shoal is needed for a successful discrimination across the boundary. Consequently, the present failure to discriminate 4 vs. 3 and 5 vs. 3 may be better explained as reflecting a limit of shoal size ratio discrimination which occurs when using the AMS. These results appear to solve the conflict with our previous findings, and seem to be in line with the idea of the existence of a single mechanism (AMS) underlying discrimination of the entire number range with a ratio limit about 2:1 in angelfish.

Interestingly, in guppies a twofold or greater change across the small-large boundary (6 vs. 3; 7 vs. 3; 9 vs. 3; 10 vs. 3) produced successful discrimination, but subjects failed with lower contrasts [27]. Data suggesting the involvement of a single mechanism of discrimination have been reported in studies with a variety of other fish species [29,31,32], as well as a diversity of other nonhuman species [8,40,51–57] and with human infants (e.g., [9,12]).

Although compelling, not all of our results point towards the existence of a single quantity discrimination system in angelfish. For example, we have found angelfish to discriminate shoals composed of 3 vs. 2 members [44], that is, a ratio of 1.5:1 that is lower than 2:1 as found here. Thus, angelfish may be sensitive not only to ratios between quantities (according to AMS) but also to absolute quantities only within the small number range (according to OFS). Interestingly, data obtained with human infants also allow this possibility. For example, it has been proposed that when the information provided by the stimulus sets is clear and reliable enough, AMS representations may take priority over the OFS [58]. In contrast, when the information from the stimuli is weak and unreliable, subjects rely on precise OFS. Thus, following this argument, in the present study the success of angelfish in the comparisons across the boundary when the shoals varied by a twofold or larger difference may indicate a reliable perception and memory of stimuli that engaged the AMS, which overrode the OFS (see also [11,59]). Results in guppies can also be explained by this hypothesis. Piffer et al. [27] showed that guppies were unable to discriminate between shoals that crossed the boundary (5 vs. 3), although, as in our current study, when the distance (ratio) between the contrasted items increased, a successful discrimination was found (6 vs. 3, 9 vs. 3). These results appear to indicate that the AMS trumps the OFS when the ratio increases above a certain threshold, thus allowing fish to discriminate shoals across the large-small boundary.

Discrimination across the large-small boundary is consequently facilitated when stimuli present redundant perceptual information, which may contribute to make a stronger signal [58]. This includes continuous information that covaries with number of items. In our present study, during the pretest period, subjects saw the full stimulus shoals moving freely. Under our conditions, swimming activity, shoal density, cumulative surface area of the stimulus shoals or of individual stimulus fish and other potential continuously varying variables were not controlled. Any of these continuous variables may contribute to the clarity of the stimulus signal, i.e., increase the information content of the stimulus employed, which may facilitate discrimination. Thus, further work is necessary to disentangle the effects of these variables under the present conditions.

Unlike in Experiment 1, in Experiment 2 when a retention interval was increased to 15 sec, angelfish showed a limited success in the cross-boundary comparisons. Given that all other experimental and procedural details were the same, we conclude that the different lengths of retention interval employed in Experiment 1 and 2 account for the observed choice performance differences between these two experiments. Although angelfish clearly demonstrated their ability to remember the prior location of the larger shoal even when a 15 sec retention interval was imposed, they were only able to discriminate between shoals across the boundary when the numerical ratio was fourfold different. These findings thus show decreased discrimination precision as the retention interval increases. Is the decreased precision due to the increased memory demand, or is it indicative of the existence of two quantity discrimination systems, AMS and OFS?

The answer to this question is somewhat speculative at this point. For example, one may argue that memory traces decay with increasing retention time, which may lead to a corresponding decrease in performance during recall. However, we do not think this may fully explain our findings. Unlike in the current study, we previously reported that angelfish could discriminate between large shoals differing by twofold during a test in which a retention interval of 15 sec was imposed [45]. These latter results indicated that angelfish were capable of meeting the high attentional demands of the task, and of acquiring and maintaining memory of the numerical size of the shoals previously seen. However, it is important to note that in this prior study [45] quantities the fish were required to discriminate did not cross the large-small boundary. Thus, our current results may not be attributable to limitations of short-term memory. More likely, they may be due to the fact that the discrimination involved crossing the large-small boundary.

Data in human infants support the idea that the AMS may be recruited in tasks involving high memory demands for tracking small sets [3,60]. Thus, in tasks of high cognitive loads, items may be represented as mental magnitudes since attentional and working memory requirements may decrease the precision of the OFS. Under such circumstances, infants have succeeded in discriminating between large and small sets. Similarly, in our current study, when the short-term memory demand of the task is increased from 2 to 15 sec, angelfish may need a clear and strong signal to discriminate between shoals, a requirement that may be satisfied by a fourfold quantity difference between the contrasted shoals but not by the smaller ratio contrasts. Thus, we speculate that angelfish use both AMS and OFS to represent shoals in the small number range, and which system they may use depends upon the circumstances and context, including signal strength as well as working memory demand, i.e., the length of time for which the contrasted items must be remembered.

## Supporting Information

S1 File Rough data of the experiments. (DOC)
