# Supplementary material for: Short-Term Memory Effects on Crossing the Boundary: Discrimination between Large and Small Quantities in Angelfish (Pterophyllum scalare)
Source: PLoS One. 2016 Sep 28;11(9):e0162923. doi: 10.1371/journal.pone.0162923 (PMC5040265; doi:10.1371/journal.pone.0162923)
Supplement: S1 File — (DOC) [file pone.0162923.s001.doc]

**Rough data of time spent close to the larger and smaller shoals and swimming activity in Experiment 1 and Experiment 2**

**Subject** **Treatment** **Group** **Smaller** **Larger** **Swimming**

1 1,00 2 vs 4 S-T 116,00 538,00 46,00 2 1,00 2 vs 4 S-T 80,00 498,00 55,00 3 1,00 2 vs 4 S-T 234,00 168,00 50,00 4 1,00 2 vs 4 S-T 350,00 235,00 29,00 5 1,00 2 vs 4 S-T 202,00 529,00 38,00 6 1,00 2 vs 4 S-T 404,00 318,00 21,00 7 1,00 2 vs 4 S-T 150,00 573,00 74,00 8 1,00 2 vs 4 S-T 503,00 306,00 37,00

9 1,00 2 vs 4 S-T 328,00 430,00 77,00 10 1,00 2 vs 4 S-T 229,00 221,00 62,00 11 1,00 2 vs 4 S-T 234,00 531,00 29,00 12 1,00 2 vs 4 S-T 201,00 580,00 83,00 13 2,00 2 vs 6 S-T 155,00 622,00 26,00 14 2,00 2 vs 6 S-T 181,00 604,00 19,00 15 2,00 2 vs 6 S-T 133,00 461,00 109,00 16 2,00 2 vs 6 S-T 361,00 348,00 83,00 17 2,00 2 vs 6 S-T 234,00 408,00 78,00 18 2,00 2 vs 6 S-T 365,00 424,00 40,00 19 2,00 2 vs 6 S-T 157,00 438,00 51,00 20 2,00 2 vs 6 S-T 289,00 433,00 54,00 21 2,00 2 vs 6 S-T 226,00 537,00 38,00

22 2,00 2 vs 6 S-T 396,00 227,00 74,00 23 2,00 2 vs 6 S-T 224,00 462,00 21,00 24 2,00 2 vs 6 S-T 233,00 546,00 14,00 25 3,00 2 vs 8 S-T 65,00 289,00 129,00 26 3,00 2 vs 8 S-T 229,00 560,00 60,00 27 3,00 2 vs 8 S-T 239,00 216,00 87,00 28 3,00 2 vs 8 S-T 402,00 267,00 74,00 29 3,00 2 vs 8 S-T 117,00 677,00 24,00 30 3,00 2 vs 8 S-T 389,00 405,00 30,00 31 3,00 2 vs 8 S-T 119,00 212,00 96,00 32 3,00 2 vs 8 S-T 189,00 515,00 49,00 33 3,00 2 vs 8 S-T 191,00 427,00 91,00 34 3,00 2 vs 8 S-T 327,00 247,00 63,00 35 3,00 2 vs 8 S-T 179,00 457,00 98,00 36 3,00 2 vs 8 S-T 246,00 504,00 22,00 37 4,00 3 vs 4 S-T 324,00 275,00 105,00 38 4,00 3 vs 4 S-T 620,00 251,00 20,00 39 4,00 3 vs 4 S-T 268,00 286,00 87,00 40 4,00 3 vs 4 S-T 161,00 190,00 73,00 41 4,00 3 vs 4 S-T 211,00 420,00 63,00 42 4,00 3 vs 4 S-T 320,00 226,00 83,00 43 4,00 3 vs 4 S-T 360,00 268,00 98,00 44 4,00 3 vs 4 S-T 338,00 290,00 54,00 45 4,00 3 vs 4 S-T 357,00 283,00 49,00 46 4,00 3 vs 4 S-T 134,00 517,00 60,00 47 4,00 3 vs 4 S-T 421,00 226,00 52,00 48 4,00 3 vs 4 S-T 332,00 390,00 68,00 49 5,00 3 vs 5 S-T 264,00 198,00 56,00 50 5,00 3 vs 5 S-T 484,00 247,00 77,00 51 5,00 3 vs 5 S-T 156,00 366,00 35,00 52 5,00 3 vs 5 S-T 204,00 225,00 89,00 53 5,00 3 vs 5 S-T 194,00 650,00 18,00 54 5,00 3 vs 5 S-T 159,00 647,00 22,00 55 5,00 3 vs 5 S-T 131,00 270,00 60,00 56 5,00 3 vs 5 S-T 592,00 186,00 25,00 57 5,00 3 vs 5 S-T 375,00 261,00 31,00 58 5,00 3 vs 5 S-T 471,00 157,00 40,00 59 5,00 3 vs 5 S-T 438,00 284,00 21,00 60 5,00 3 vs 5 S-T 267,00 559,00 19,00 61 6,00 3 vs 6 S-T 116,00 679,00 33,00 62 6,00 3 vs 6 S-T 373,00 424,00 40,00 63 6,00 3 vs 6 S-T 131,00 669,00 23,00 64 6,00 3 vs 6 S-T 244,00 517,00 26,00 65 6,00 3 vs 6 S-T 344,00 374,00 55,00 66 6,00 3 vs 6 S-T 481,00 320,00 24,00 67 6,00 3 vs 6 S-T 480,00 276,00 31,00 68 6,00 3 vs 6 S-T 236,00 524,00 34,00 69 6,00 3 vs 6 S-T 189,00 575,00 23,00 70 6,00 3 vs 6 S-T 394,00 255,00 73,00 71 6,00 3 vs 6 S-T 168,00 612,00 33,00 72 6,00 3 vs 6 S-T 127,00 636,00 81,00 73 7,00 3 vs 7 S-T 308,00 423,00 65,00 74 7,00 3 vs 7 S-T 308,00 361,00 51,00 75 7,00 3 vs 7 S-T 296,00 299,00 78,00 76 7,00 3 vs 7 S-T 102,00 450,00 98,00 77 7,00 3 vs 7 S-T 232,00 559,00 20,00 78 7,00 3 vs 7 S-T 116,00 679,00 19,00 79 7,00 3 vs 7 S-T 280,00 375,00 85,00 80 7,00 3 vs 7 S-T 275,00 533,00 22,00 81 7,00 3 vs 7 S-T 393,00 233,00 32,00 82 7,00 3 vs 7 S-T 170,00 548,00 49,00 83 7,00 3 vs 7 S-T 262,00 425,00 89,00 84 7,00 3 vs 7 S-T 395,00 470,00 18,00 85 8,00 3 vs 9 S-T 118,00 567,00 69,00 86 8,00 3 vs 9 S-T 234,00 254,00 77,00 87 8,00 3 vs 9 S-T 218,00 271,00 48,00 88 8,00 3 vs 9 S-T 87,00 505,00 33,00 89 8,00 3 vs 9 S-T 171,00 548,00 102,00 90 8,00 3 vs 9 S-T 211,00 203,00 111,00 91 8,00 3 vs 9 S-T 230,00 335,00 65,00 92 8,00 3 vs 9 S-T 130,00 607,00 36,00 93 8,00 3 vs 9 S-T 146,00 499,00 31,00 94 8,00 3 vs 9 S-T 160,00 582,00 30,00 95 8,00 3 vs 9 S-T 307,00 468,00 27,00 96 8,00 3 vs 9 S-T 148,00 584,00 38,00

**Exp. 2**

97 9,00 2 vs 4 T 203,00 507,00 34,00 98 9,00 2 vs 4 T 396,00 394,00 24,00 99 9,00 2 vs 4 T 603,00 91,00 22,00 100 9,00 2 vs 4 T 263,00 380,00 18,00 101 9,00 2 vs 4 T 211,00 377,00 56,00 102 9,00 2 vs 4 T 252,00 288,00 87,00 103 9,00 2 vs 4 T 122,00 285,00 33,00 104 9,00 2 vs 4 T 381,00 186,00 30,00 105 9,00 2 vs 4 T 423,00 459,00 11,00 106 9,00 2 vs 4 T 575,00 269,00 22,00 107 9,00 2 vs 4 T 261,00 300,00 84,00 108 9,00 2 vs 4 T 527,00 157,00 52,00 109 10,00 2 vs 6 T 606,00 226,00 21,00 110 10,00 2 vs 6 T 50,00 674,00 37,00 111 10,00 2 vs 6 T 408,00 175,00 40,00 112 10,00 2 vs 6 T 152,00 638,00 26,00 113 10,00 2 vs 6 T 53,00 385,00 71,00 114 10,00 2 vs 6 T 324,00 152,00 52,00 115 10,00 2 vs 6 T 88,00 595,00 46,00 116 10,00 2 vs 6 T 183,00 550,00 34,00 117 10,00 2 vs 6 T 545,00 196,00 23,00 118 10,00 2 vs 6 T 328,00 78,00 26,00 119 10,00 2 vs 6 T 231,00 325,00 53,00 120 10,00 2 vs 6 T 329,00 294,00 33,00 121 11,00 2 vs 8 T 69,00 698,00 22,00 122 11,00 2 vs 8 T 332,00 505,00 21,00 123 11,00 2 vs 8 T 114,00 605,00 24,00 124 11,00 2 vs 8 T 96,00 699,00 26,00 125 11,00 2 vs 8 T 151,00 596,00 35,00 126 11,00 2 vs 8 T 183,00 590,00 25,00 127 11,00 2 vs 8 T 212,00 548,00 27,00 128 11,00 2 vs 8 T 101,00 694,00 18,00 129 11,00 2 vs 8 T 160,00 567,00 45,00 130 11,00 2 vs 8 T 151,00 509,00 36,00 131 11,00 2 vs 8 T 105,00 622,00 29,00 132 11,00 2 vs 8 T 440,00 281,00 34,00 133 12,00 3 vs 4 T 173,00 163,00 35,00 134 12,00 3 vs 4 T 201,00 383,00 75,00 135 12,00 3 vs 4 T 165,00 569,00 23,00 136 12,00 3 vs 4 T 271,00 370,00 51,00 137 12,00 3 vs 4 T 326,00 349,00 44,00 138 12,00 3 vs 4 T 355,00 76,00 88,00 139 12,00 3 vs 4 T 197,00 223,00 90,00 140 12,00 3 vs 4 T 150,00 566,00 37,00 141 12,00 3 vs 4 T 330,00 207,00 68,00 142 12,00 3 vs 4 T 190,00 517,00 45,00 143 12,00 3 vs 4 T 221,00 423,00 83,00 144 12,00 3 vs 4 T 336,00 153,00 66,00 145 13,00 3 vs 5 T 230,00 412,00 62,00 146 13,00 3 vs 5 T 160,00 282,00 79,00 147 13,00 3 vs 5 T 566,00 204,00 28,00 148 13,00 3 vs 5 T 178,00 338,00 94,00 149 13,00 3 vs 5 T 463,00 177,00 48,00 150 13,00 3 vs 5 T 214,00 208,00 114,00 151 13,00 3 vs 5 T 686,00 125,00 22,00 152 13,00 3 vs 5 T 251,00 480,00 40,00 153 13,00 3 vs 5 T 139,00 344,00 41,00 154 13,00 3 vs 5 T 368,00 128,00 65,00 155 13,00 3 vs 5 T 109,00 535,00 87,00 156 13,00 3 vs 5 T 281,00 332,00 100,00 157 14,00 3 vs 6 T 322,00 335,00 44,00 158 14,00 3 vs 6 T 234,00 435,00 33,00

159 14,00 3 vs 6 T 67,00 719,00 38,00 160 14,00 3 vs 6 T 279,00 515,00 16,00 161 14,00 3 vs 6 T 273,00 319,00 62,00 162 14,00 3 vs 6 T 195,00 568,00 24,00 163 14,00 3 vs 6 T 418,00 264,00 90,00 164 14,00 3 vs 6 T 228,00 500,00 25,00 165 14,00 3 vs 6 T 267,00 399,00 49,00 166 14,00 3 vs 6 T 300,00 389,00 56,00 167 14,00 3 vs 6 T 618,00 139,00 24,00 168 14,00 3 vs 6 T 380,00 345,00 87,00 169 15,00 3 vs 7 T 298,00 306,00 17,00 170 15,00 3 vs 7 T 522,00 288,00 26,00 171 15,00 3 vs 7 T 516,00 185,00 49,00 172 15,00 3 vs 7 T 18,00 667,00 28,00 173 15,00 3 vs 7 T 549,00 269,00 47,00 174 15,00 3 vs 7 T 253,00 308,00 91,00 175 15,00 3 vs 7 T 349,00 318,00 81,00 176 15,00 3 vs 7 T 85,00 506,00 103,00 177 15,00 3 vs 7 T 92,00 673,00 35,00 178 15,00 3 vs 7 T 245,00 513,00 26,00 179 15,00 3 vs 7 T 281,00 434,00 41,00 180 15,00 3 vs 7 T 102,00 555,00 42,00 181 16,00 3 vs 9 T 327,00 165,00 51,00 182 16,00 3 vs 9 T 110,00 704,00 21,00 183 16,00 3 vs 9 T 66,00 671,00 22,00 184 16,00 3 vs 9 T 247,00 540,00 29,00 185 16,00 3 vs 9 T 213,00 139,00 42,00 186 16,00 3 vs 9 T 417,00 197,00 33,00 187 16,00 3 vs 9 T 68,00 558,00 37,00 188 16,00 3 vs 9 T 95,00 464,00 40,00 189 16,00 3 vs 9 T 603,00 183,00 28,00 190 16,00 3 vs 9 T 159,00 625,00 29,00 191 16,00 3 vs 9 T 238,00 352,00 64,00 192 16,00 3 vs 9 T 147,00 457,00 56,00 193 17,00 3 vs 12 T 152,00 654,00 16,00 194 17,00 3 vs 12 T 193,00 541,00 55,00 195 17,00 3 vs 12 T 220,00 468,00 51,00 196 17,00 3 vs 12 T 409,00 255,00 55,00 197 17,00 3 vs 12 T 100,00 695,00 52,00 198 17,00 3 vs 12 T 247,00 479,00 72,00 199 17,00 3 vs 12 T 314,00 331,00 55,00 200 17,00 3 vs 12 T 54,00 691,00 30,00 201 17,00 3 vs 12 T 333,00 389,00 94,00 202 17,00 3 vs 12 T 386,00 421,00 76,00 203 17,00 3 vs 12 T 177,00 683,00 12,00 204 17,00 3 vs 12 T 131,00 665,00 19,00
